# Exposure to Silver Nanospheres Leads to Altered Respiratory Mechanics and Delayed Immune Response in an *in Vivo* Murine Model

**DOI:** 10.3389/fphar.2018.00213

**Published:** 2018-03-26

**Authors:** Danielle Botelho, Bey F. Leo, Christopher Massa, Srijata Sarkar, Terry Tetley, Kian F. Chung, Shu Chen, Mary P. Ryan, Alexandra Porter, Elena N. Atochina-Vasserman, Junfeng Zhang, Stephan Schwander, Andrew J. Gow

**Affiliations:** ^1^Department of Pharmacology and Toxicology, Rutgers University, Piscataway, NJ, United States; ^2^Department of Materials and London Centre for Nanotechnology, Imperial College London, London, United Kingdom; ^3^Nanotechnology and Catalysis Research Center, University of Malaya, Kuala Lumpur, Malaysia; ^4^Department of Environmental and Occupational Health, School of Public Health, Rutgers University, Piscataway, NJ, United States; ^5^National Heart and Lung Institute, Imperial College London, London, United Kingdom; ^6^RASA Center in Tomsk, Tomsk Polytechnic University, Tomsk, Russia; ^7^RASA Center, Kazan Federal University, Kazan, Russia; ^8^Nicholas School of the Environment, Duke University, Durham, NC, United States

**Keywords:** silver nanoparticles, lung, pulmonary function, surfactant, inflammation, nanoparticles

## Abstract

Here we examine the organ level toxicology of both carbon black (CB) and silver nanoparticles (AgNP). We aim to determine metal-specific effects to respiratory function, inflammation and potential interactions with lung lining fluid (LLF). C57Bl6/J male mice were intratracheally instilled with saline (control), low (0.05 μg/g) or high (0.5 μg/g) doses of either AgNP or CB 15 nm nanospheres. Lung histology, cytology, surfactant composition and function, inflammatory gene expression, and pulmonary function were measured at 1, 3, and 7 days post-exposure. Acutely, high dose CB resulted in an inflammatory response, increased neutrophilia and cytokine production, without alteration in surfactant composition or respiratory mechanics. Low dose CB had no effect. Neither low nor high dose AgNPs resulted in an acute inflammatory response, but there was an increase in work of breathing. Three days post-exposure with CB, a persistent neutrophilia was noted. High dose AgNP resulted in an elevated number of macrophages and invasion of lymphocytes. Additionally, AgNP treated mice displayed increased expression of IL1B, IL6, CCL2, and IL10. However, there were no significant changes in respiratory mechanics. At day 7, inflammation had resolved in AgNP-treated mice, but tissue stiffness and resistance were significantly decreased, which was accompanied by an increase in surfactant protein D (SP-D) content. These data demonstrate that the presence of metal alters the response of the lung to nanoparticle exposure. AgNP-surfactant interactions may alter respiratory function and result in a delayed immune response, potentially due to modified airway epithelial cell function.

## Introduction

Silver nanoparticles (AgNPs) are increasingly utilized in consumer products, and are valued for their antimicrobial properties (Wang et al., 2005, 2014; Royce et al., 2014). As consumer and occupational exposures increase, with inhalation as a primary route of exposure (Royce et al., 2014), it is necessary to study potential consequences of AgNP exposure *in vivo* and their effects on respiratory function and inflammation. To determine effects specific to the metal properties of AgNPs, we compared exposure to non-metal CB.

Many previous studies have focused on the *in vitro* interactions of metal NPs and lung lining proteins, lipids and cells (Bakshi et al., 2008; Herzog et al., 2009, 2014; Richter et al., 2011; Comfort et al., 2014). The study of gold NPs in the presence of dipalmitoyl phosphatidylcholine (DPPC), palmitoyl-oleoyl-phosphatidylglycerol, and surfactant protein B (SP-B) demonstrated that the metal NPs become coated by lipids and form aggregated strands (Bakshi et al., 2008). This coating and consequent aggregation led to increased surface tension measurements, indicating potential for increased work of breathing when metal NPs were inhaled (Bakshi et al., 2008). Herzog et al. (2014) used AgNPs and a complex triple cell culture with an air-liquid interface to mimic the conditions of the lung (Herzog et al., 2014). Data from this study indicated differential particle interactions depending upon AgNP interactions at the air-liquid interface vs. AgNPs submerged in the liquid phase. AgNPs aggregated with cells at the air-liquid interface, but did not trigger a cytotoxic response, while AgNPs immersed in the liquid phase led to cytotoxicity. Findings from these previous *in vitro* studies indicate potential for pronounced mechanical and inflammatory responses by the lung due to metal NP exposure. Yet, while these studies closely mimic respiratory conditions, the complex organ level toxicology must be assessed *in vivo* for NP exposures.

Our previous *in vivo* studies examining organ level toxicology of AgNPs (with gold cores) have demonstrated both size- and stabilization-dependent lung function effects (Botelho et al., 2015). Usually, metal NPs are stabilized by a “capping-agent” such as citrate or polyvinylpyrrolidone (PVP) (Bakshi et al., 2008; Leo et al., 2013). We have demonstrated previously that citrate-stabilized AgNPs elicit little biological response acutely in comparison to PVP-stabilized counterparts (Botelho et al., 2015). In this current study we use CB as a control for metal effects. A CB nanosphere has a Buckminster Fullerene structure, while AgNPs are stabilized using citrate, reduced using sodium borohydride and are redox-reactive (Leo et al., 2013). These differences in surface composition allow for differential interactions with components of the lung lining fluid (LLF).

In this study, we have taken citrate-stabilized AgNPs of solid silver composition to determine respiratory effects over the course of 7 days. We hypothesized that AgNPs would aggregate and interact with the lipid portion of the LLF in a divergent manner compared to the non-metal control we used, CB. Further, we proposed that metal NPs, by interacting with the lipids of the LLF and through their differential solution characteristics, would increase respiratory effort and activate divergent inflammatory pathways from non-metal NP.

## Methods

### Particle specifications

Carbon black and AgNPs (15 nm) were designed and characterized by the Department of Materials and London Center for Nanotechnology, Imperial College London (Leo et al., 2013). AgNPs were stabilized using citrate and reduced using sodium borohydride (Leo et al., 2013). The detailed physiochemical properties of these materials have been previously described (Hu et al., 2014).

### Animal model and instillation

Nine-week-old C57-BL6 Jackson wild-type male mice were intratracheally instilled with either Hank's Balanced Salt Solution (HBSS), silver nitrate solution, CB or AgNPs following anesthesia using a ketamine/xylazine combination. 0.05 or 0.5 μg particle/g body weight were suspended into HBSS to a final volume of 50 μL. Probe sonication of NPs was utilized immediately before intratracheal instillation. Mice given 0.5 μg of CB or AgNP were examined at 1, 3, or 7 days post-instillation. Low dose particle treated mice (0.05 μg/g body weight) were only examined 1 day post-instillation. Silver nitrate instillations were given at a high dose (0.5 μg/g body weight) and mice were examined only at 7 days post-instillation. At examination mice were anesthetized and attached to a ventilator in order to assess mechanical function (see below). Following measurement of lung function, bronchoalveolar lavage and tissues were harvested and analyzed as described below.

This protocol was approved by the Rutgers University Institutional Animal Care and Use Committee (IACUC) (Protocol Number: 06-028). The study was conducted in accordance with the recommendations in the Guide for the Care and Use of Laboratory Animals of the National Institutes of Health. Intratracheal instillations and mechanical ventilation were conducted under ketamine/xylazine anesthesia, and all efforts were made to minimize suffering. Animals were sacrificed using a lethal dose of ketmine/xylazine and exsanguination.

### Bronchoalveolar lavage

A bronchoalveolar lavage of the whole lung was performed using 10 mM HEPES buffered saline in 1 mL increments four times (prior to inflation fixing with paraformaldehyde). Bronchoalveolar lavage fluid (BALF) was centrifuged in order to obtain a cell pellet and the cell free supernatant. The cell-free BALF was collected for protein and phospholipid assays. The BALF cells were re-suspended in 1 mL buffered saline for cell count, cytology and RT-PCR analysis (Casey et al., 2005; Atochina-Vasserman et al., 2009; Groves et al., 2012; Botelho et al., 2015).

### Histology

Using a 3% paraformaldehyde/2% sucrose solution, the left lung was inflation-fixed and then embedded in paraffin, sectioned and stained with hematoxylin and eosin (H&E). The Olympus VS120-SL Virtual Slide System was used for imaging lung sections (Atochina-Vasserman et al., 2009; Groves et al., 2012; Botelho et al., 2015).

### Cell count and cell differential

Using the Beckman Coulter^TM^ Multisizer^TM^ 3 Coulter Counter®, the cell number from BALF samples was assessed. Approximately 30,000 cells were centrifuged onto a glass slide at 300 × g for 10 min, air dried, and stained with Diff-Quik buffered modified Wright-Giemsa stain. Cell differentials were assessed manually using a light microscope (Casey et al., 2005; Atochina-Vasserman et al., 2009; Groves et al., 2012; Botelho et al., 2015).

### Real-time polymerase chain reaction (RT-PCR)

RNA was prepared from BALF cells using a QIAshredderTM kit and converted to cDNA. The cell samples for each treatment group were pooled into one larger sample for analysis. Thermocycling was used to analyze mRNA expression of CCL2, IL1B, IL6, CXCL10, IL10, and IL12B in the treated mice. Fold expression was calculated by the ΔΔCT method normalizing to the HBSS (control) treatment group (Sarkar et al., 2012).

### Protein and phospholipid assay

Whole BALF protein concentration was determined using a Low Protein BCA Assay kit by Lamda Biotech, Inc. To estimate phospholipid content whole BALF was fractionated into small and large aggregate portions by centrifugation at 20,000 × g for 1 h at 4°C. The supernatant is the protein-rich small aggregate fraction, while the pellet can be re-suspended in a small volume of saline (35 μl) and is the lipid-rich large aggregate fraction. Inorganic phosphate from the lipid-rich fractions was measured as an estimate of the phospholipid content (Bligh and Dyer, 1959; Atochina-Vasserman et al., 2009; Botelho et al., 2015).

### Immunoblotting and densitometry

To determine surfactant protein content reducing NuPAGE was performed using individual BALF samples from treated mice. Gels were transferred to BioRad Immun-blot® PVDF membranes, incubated with SP-D (from M.Beers University of Pennsylvania) or SP-B (from a Pastva Duke University) antibody and goat anti-rabbit antibody linked to horseradish peroxidase (Bio-rad), and imaged using Amersham^TM^ ECL^TM^ Prime Western Blotting Detection Reagent. Densitometry was performed to quantify the chemiluminescent signal. Whole BALF was used for SP-D analysis and sample load was normalized to volume. Large aggregate BALF was used for SP-B analysis and sample load was normalized to phospholipid content as determined by the phospholipid assay (Atochina-Vasserman et al., 2009; Botelho et al., 2015).

### Capillary surfactometry

Surface tension was measured using a capillary surfactometer by Calmia Medical, Inc. Samples were loaded at a consistent phospholipid concentration of 1.0 μg phospholipid/μl. Following sample loading, compressed air was applied to the capillary at increasing pressures. Both the initial pressure to disrupt the large aggregate BALF and percent capillary open over the course of 2 min was recorded (Atochina-Vasserman et al., 2009; Botelho et al., 2015).

### Mechanical ventilation and respiratory model

One, three, and seven days following particle instillation, mice were anesthetized and ventilated using the flexiVent (SCIREQ, Montreal, Canada) at positive end expiratory pressures (PEEPs) (0, 1, 3, and 6 cmH2O). Mechanical ventilation with forced oscillation maneuvers was used to measure impedance [Z(f) = P(f)/ V(f)] (Moriya et al., 2003). Output impedance spectra were used to generate resistance (R_L_) and elastance (E_L_) spectra across the frequency range, and fit to a model of heterogeneous lung function (Groves et al., 2012, 2013; Massa et al., 2014; Botelho et al., 2015):

$$\begin{array}{l} {\text{R}}_{\text{L}}=\left(\text{a}+\text{b}f\right)/\left(\text{c}+f\right)\text{ and }{\text{E}}_{\text{L}}=\text{E}0+\Delta \text{E}\left(1-{\text{e}}^{-\beta \text{f}}\right) \end{array}$$

### Statistical methods

Outcome data were analyzed via two-way analysis of variance (ANOVA) across dose and particle administered. Pairwise comparisons were made using protected *t*-test and a significance level of *p* < 0.05. Animal numbers per group were 4–6. For lung function data, model fits were estimated for each treatment group as well as the parameters of the R_L_ and E_L_ curves as previously demonstrated (Groves et al., 2012, 2013; Massa et al., 2014; Botelho et al., 2015). A two-way analysis of variance (ANOVA) was used to determine significant differences in the mean value of each parameter for treatment groups and PEEP. Pairwise comparisons were made using the Holm-Sidak method.

## Results

### 1 Day post-instillation

**Figure 1:**
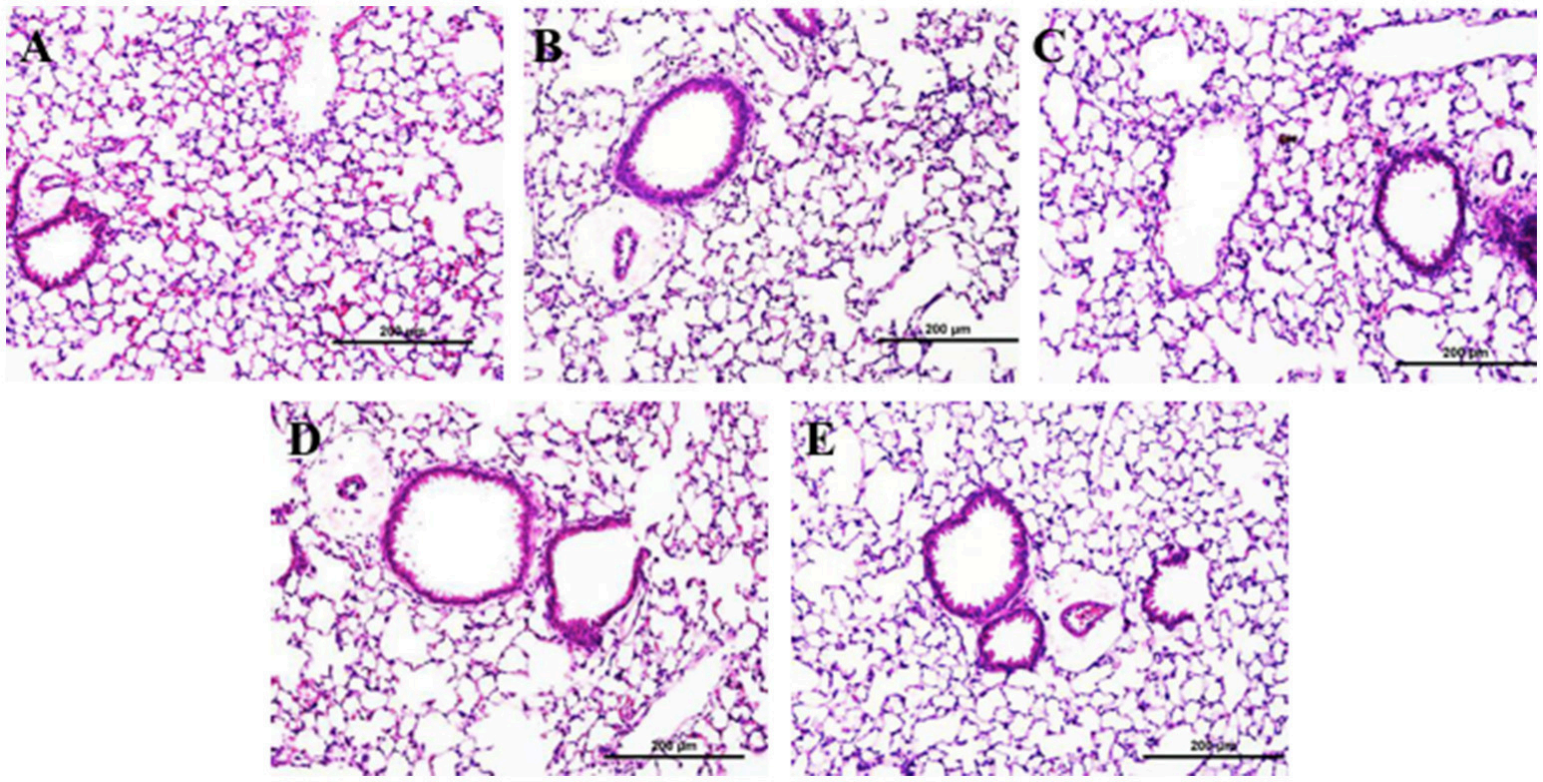
Lung tissue histology 1 day post-particle instillation. Inflation fixed paraffin embedded sections were stained with hematoxylin and eosin. **(A)** Control (HBSS) **(B)** Low dose CB **(C)** High dose CB **(D)** Low dose AgNPs **(E)** High dose AgNPs; histology does not show any significant injury associated with particle treatment (Enlarged H&E images 20x).

**Figure 2:**
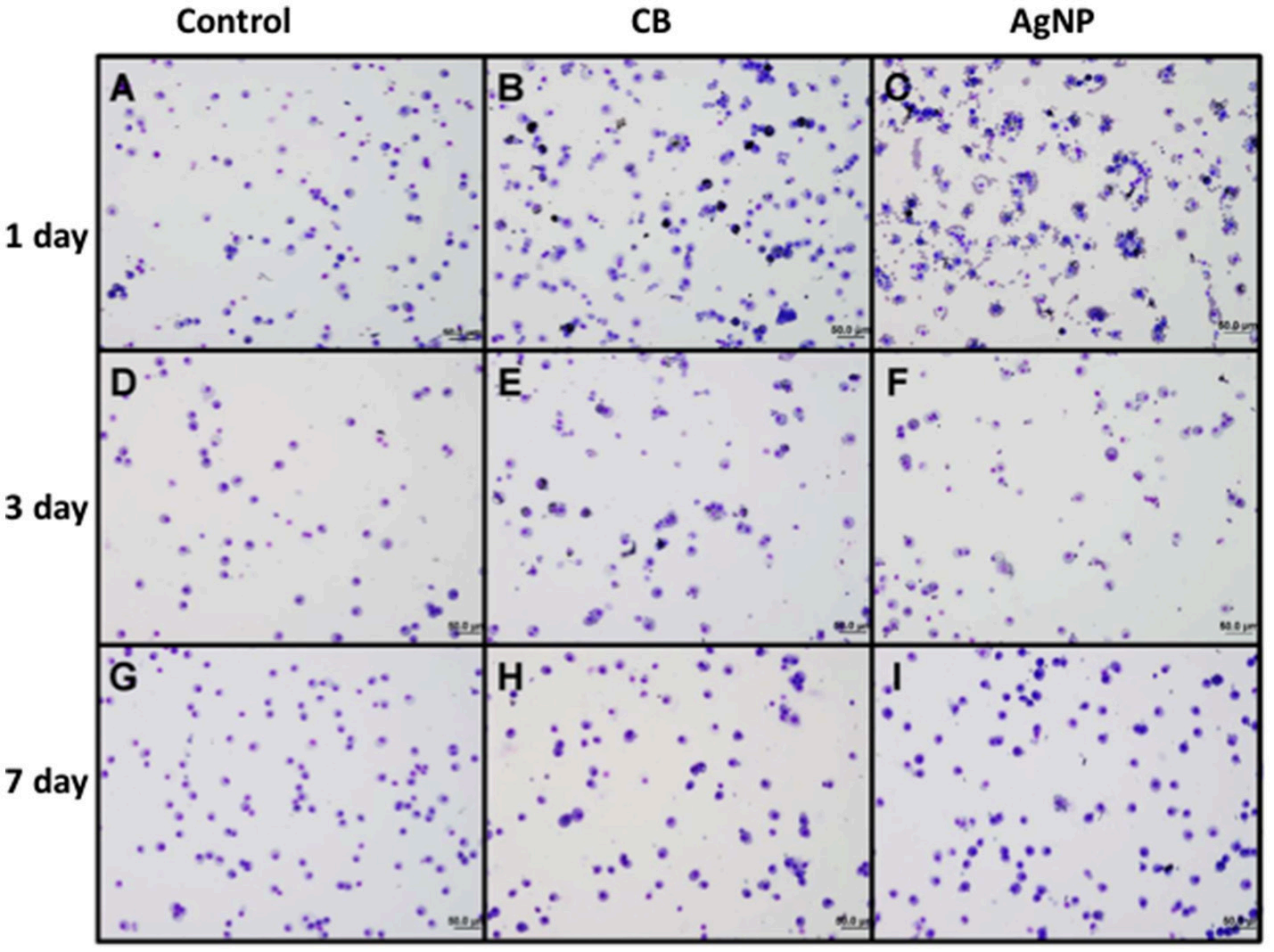
Cytology of BAL cells following administration of high dose NPs. **(A,D,G)** Control (HBSS); **(B,E,H)** High dose CB; **(C,F,I)** High dose AgNPs; **(A–C)** One day **(D–F)** Three days and **(G–I)** Seven days post-installion; acutely activated macrophages are observed following both NP treatments, with uptake of CB into macrophages being visible. No visible CB was observed in macrophages seven days after instillation (images are displayed at 20x).

**Figure 3:**
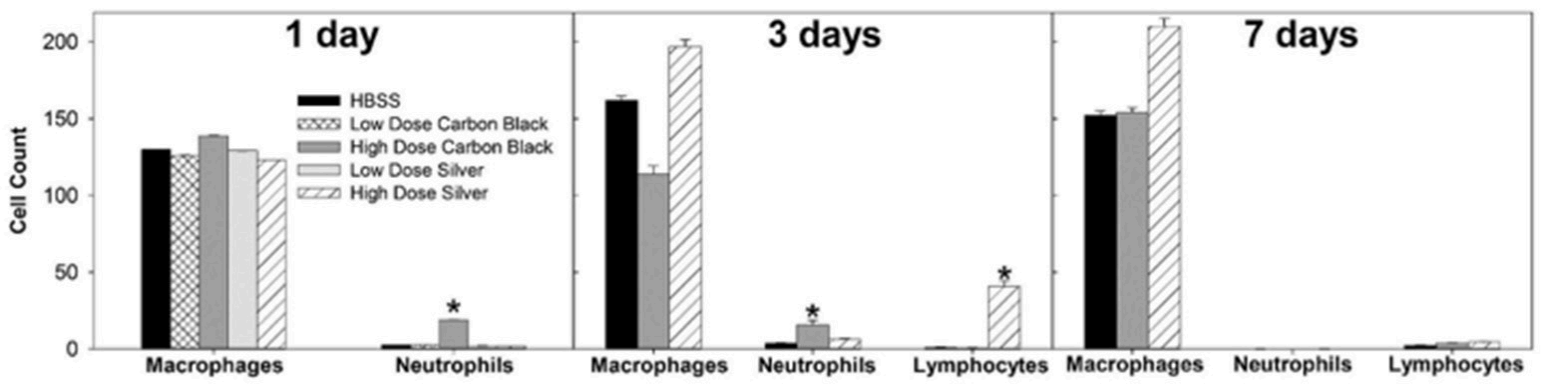
Cell differentials. At one day post-instillation, high dose CB-treatment results in neutrophilia. Three Days post-instillation CB-mediated neutrophilia begins to resolve, while high dose silver treatment results in increased macrophages and lymphocytes. Seven Days post-instillation, persisting elevated macrophages are seen with silver particles, while neutrophils and lymphocytes have resolved (^*^p < 0.05 compared to the control for that cell type).

**Figure 4:**
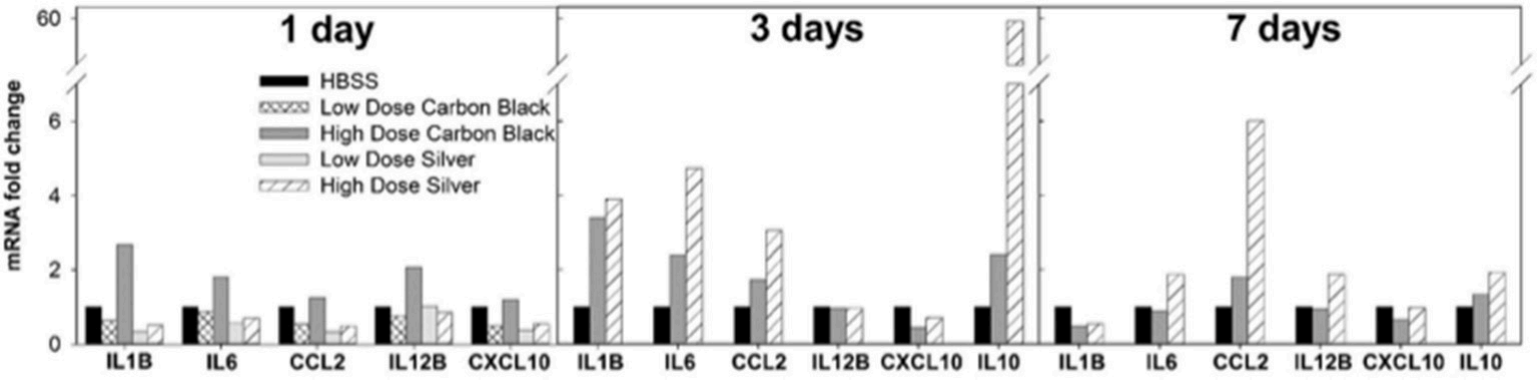
BAL cell mRNA expression by PCR. One day post-instillation high dose CB-treated mice demonstrate increased expression of IL-1β, IL-6, and IL-12B as compared to control (HBSS). Three days post-instillation high dose CB-treated mice persist with elevated IL-1β and IL-6 expression, while AgNP-treated mice demonstrate increased expression of IL-1β, IL-6, CCL2, and IL-10. Seven days post-instillation all inflammatory markers have resolved in CB-treated mice, but in AgNP-treated mice CCL2 remains elevated. (Samples were pooled for each treatment group at each time point, *n* = 4–6 mice).

There was no evidence of injury in any of the saline (control) or particle-treated mice as assessed by histology (Figure 1). Cytological examination, however, revealed a significant increase of neutrophils in lungs of high dose CB particle-treated mice, as well as obvious uptake of CB into macrophages (Figures 2, 3). This indicates activation of an inflammatory response, which is supported by the PCR data where there was an increase in expression of *IL1B, IL6*, and *IL12B* as compared to the control (Figure 4); this cytokine expression profile confirms macrophage activation. However, there was no invasion of inflammatory cells or inflammatory activation indicated by way of cytology or PCR for low dose CB or for low or high dose AgNP-treated mice.

**Figure 5:**
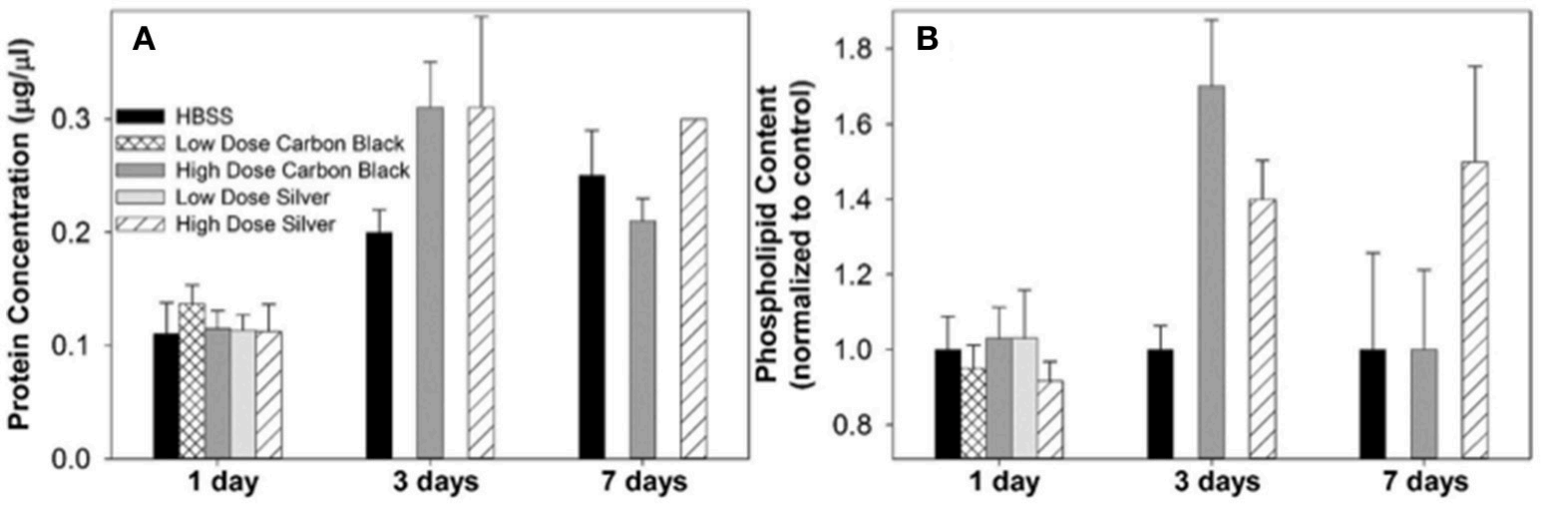
Protein concentration and phospholipid content in BAL. **(A)** At each time point, treatment groups were not significantly different from control (HBSS). However, at three days post-instillation all groups displayed an increase in BAL protein concentration which began to resolve by seven days in CB-treated mice **(B)** There was no significant difference between treatment groups at any time point, however at three days post-instillation NP treated mice displayed increases in phospholipid content as compared to the control. While this increase resolved by seven days in CB-treated mice, AgNP-treated mice continued to display increases in phospholipid content as compared to the control.

Additionally, there were no significant differences between treatment groups in protein concentration, phospholipid content, SP-D/B ratios, or capillary surfactometry at 1 day post-instillation (Figure 5, Figure S2, and Table S1). Although there was an inflammatory response elicited by high doses of CB NPs, there was no significant difference in respiratory mechanics (resistance or elastance) between CB-treated mice (low or high dose) as compared to the saline control (**Figure 7**). Furthermore, despite a lack of inflammatory response in AgNP-treated mice (low and high dose) demonstrated a significant increase in tissue stiffness as compared to the control; but no significant differences in airway or tissue resistance (**Figure 7**, low dose AgNP results not shown). These significant increases in elastance were restricted to the low frequency end of the spectra indicating changes in the parenchyma.

### 3 Days post-instillation

Three days post-single instillation, histology revealed no signs of injury to the lung as a result of particle exposure (Figure S1). Cytology confirmed persisting neutrophilia in high dose CB-treated mice and continued uptake of CB particle in macrophages (Figures 2,3). Additionally, cytospins of BAL fluid from high dose AgNP-treated mice demonstrated an increase of lymphocytes and macrophages (Figure 2), indicating activation of an inflammatory response in both particle- treated groups, but by potentially different pathways. PCR demonstrated continued elevated expression of *IL1B* and *IL6* as well as an increase in *CCL2* and *IL10* for high dose CB particle-treated mice indicating activation of macrophages, a response to inflammation, and recruitment of monocytes (Figure 4). While these same cytokines were expressed in AgNP-treated mice, there was also a 60-fold increase in *IL10* gene expression (Figure 4). IL10 is produced by monocytes and lymphocytes, but it is the magnitude of expression of IL10 in AgNP-treated mice as compared to CB-treated mice coupled with lymphocyte invasion for AgNP-treated mice that indicates a significant difference in inflammatory profile of the two groups.

**Figure 6:**
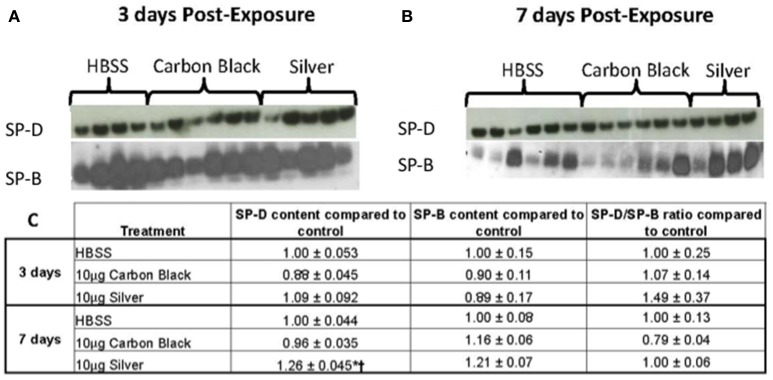
Surfactant protein immunblotting. **(A)** SP-D (top) and SP-B (bottom) three days-post-instillation **(B)** SP-D (top) and SP-B (bottom) seven days-post-instillation **(C)** Table of densitometry values for SP-D and SP-B content and SP-D/SP-B ratios normalized to the control. No differences in SP-D content until seven days post-instillation (^*^p < 0.001 compared to the control, ^†^*p* < 0.001 compared to CB treatment).

There was no significant difference in protein concentration between groups (Figure 5), consistent with a lack of damage to barrier function as a result of lung injury; however, there was a significant increase in phospholipid content for CB-treated mice as compared to the control (Figure 5). Despite changes in inflammatory profile for AgNP-treated mice and persisting inflammation, coupled with a change in phospholipid content for CB-treated mice there were no significant differences between groups for SP-D/SP-B ratios or capillary surfactometry (Figure 6 and Table S1). Respiratory mechanics analysis revealed no significant differences in elastance or resistance between treatment groups, indicating resolution of the tissue stiffness previously demonstrated by AgNP-treated mice at 1 day post-instillation.

### 7 Days post-instillation

**Figure 7:**
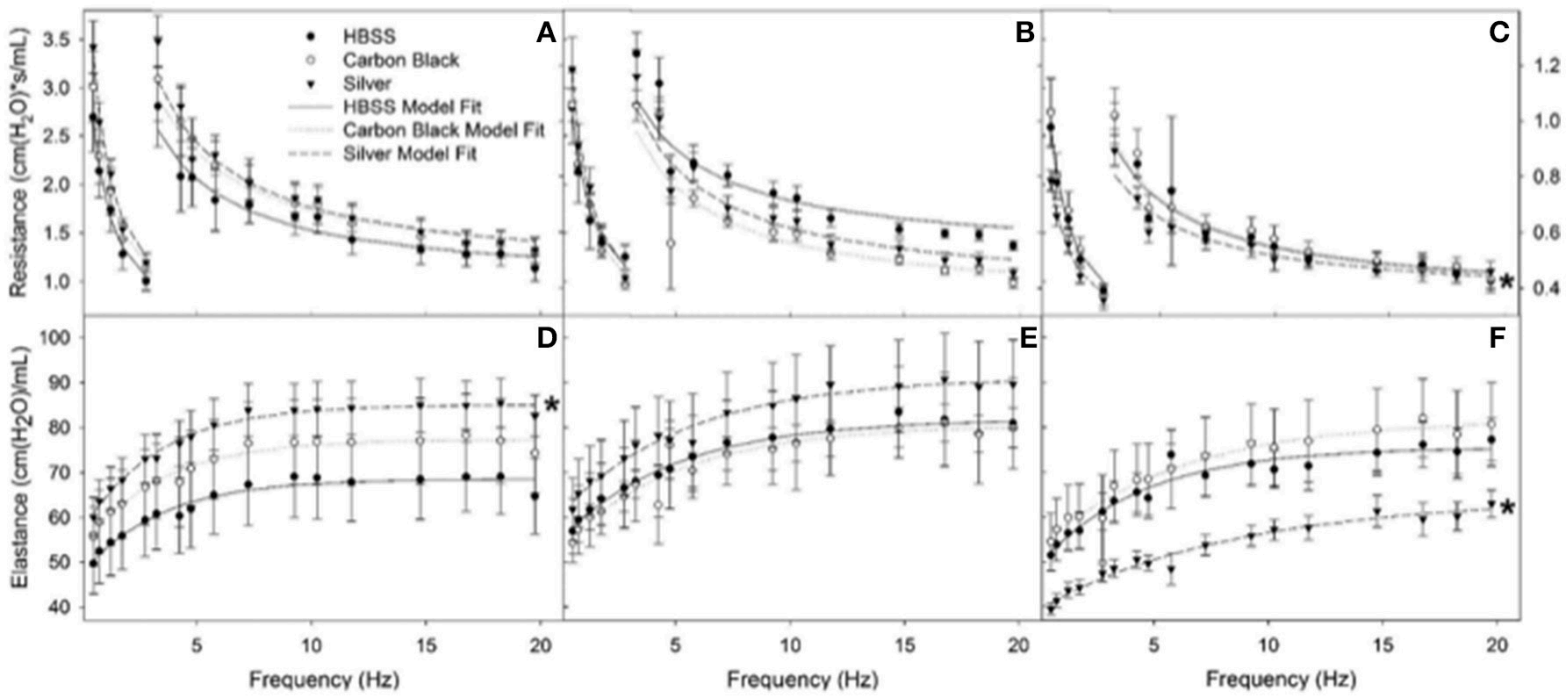
Resistance (RL) and elastance (EL) spectra using forced oscillation technique. Analysis of impedance measurements at PEEP 3 **(A)** HBSS RL spectra **(B)** CB RL spectra **(C)** AgNP RL spectra **(D)** HBSS EL spectra **(E)** CB EL spectra **(F)** AgNP EL spectra at 1, 3, 7 days. Both HBSS and High Dose CB treated mice did not significantly differ in elastance or resistance across time points within their treatment groups or when compared to one another. AgNP-treated mice displayed acute tissue stiffness. At seven days post-instillation, AgNP-treated mice experience significantly decreased elastance (decreased tissue stiffness), as well as significantly decreased resistance (as compared to silver treated mice at one and three days post-instillation, as well as compared to all other treatments at all other time points). At one day post-instillation low dose particle treatments demonstrated no significant differences as compared to the control. ^*^represents significantly different from HBSS (*p* < 0.05).

**Figure 8:**
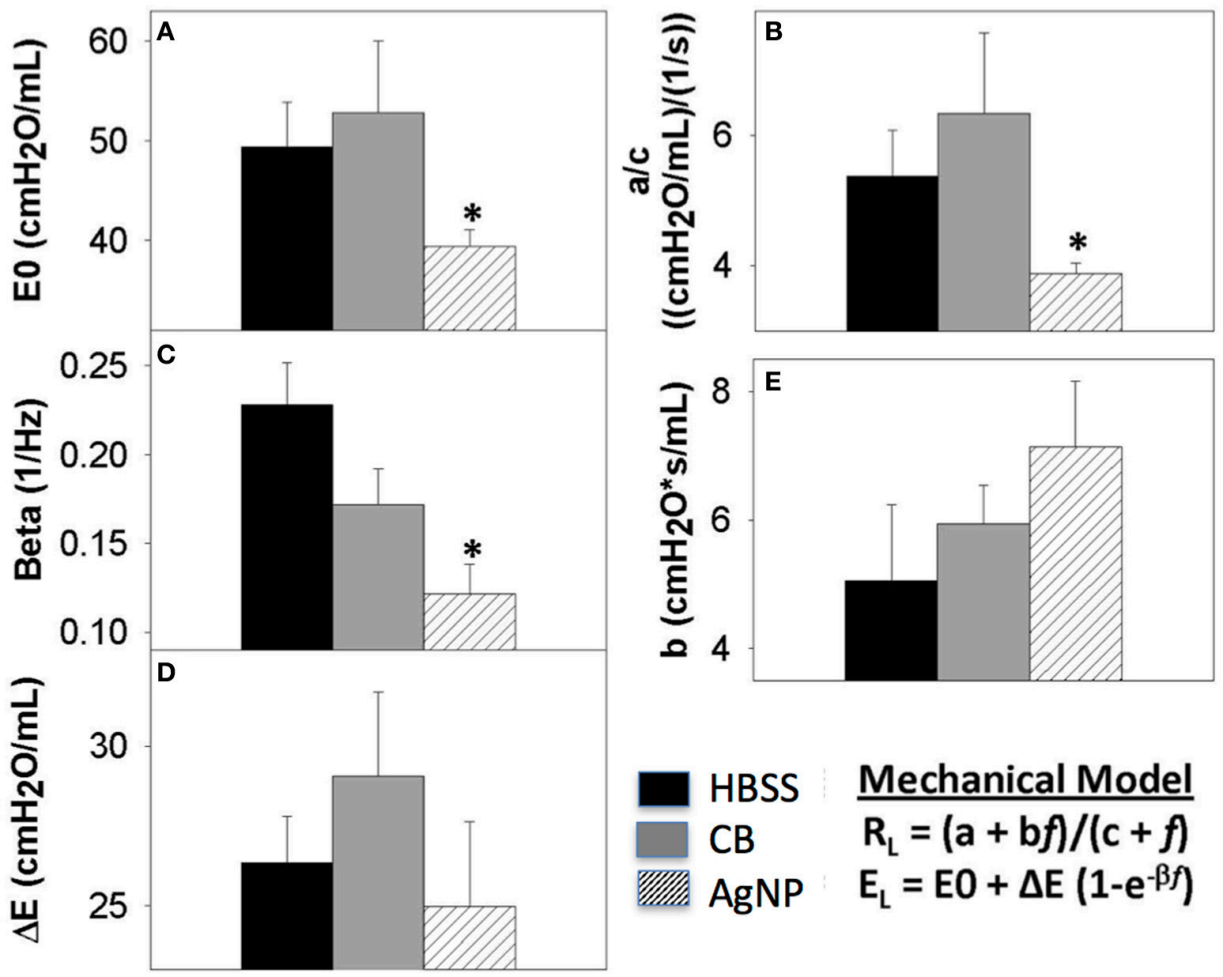
Seven days post-exposure resistance (RL) and elastance (EL) model parameters. Analysis of model parameters at PEEP 3 **(A)** inherent tissue stiffness (E0) **(B)** Low frequency resistance (a/c) **(C)** rate of change of elastance (beta) **(D)** magnitude of change in elastance (ΔE) **(E)** high frequency resistance (b). The greatest changes in spectra were seen at seven days, and these parameters are extracted from the model for HBSS, High Dose CB, and High Dose AgNP. The most significant changes occur in the low frequency end of the spectra, where AgNP-treated mice display a significantly decreased inherent tissue stiffness and resistance in the lower airways as compared to the control. These differences are not resolved with increasing PEEP. (^*^p < 0.05 compared to the control).

At 7 days post single instillation there was continuing lack of sign of lung injury by histology (Figure S2). As evidenced by cytology, there was near complete resolution of invading neutrophils and lymphocytes for both CB- and AgNP-treated mice (Figure 2). PCR indicated near-complete resolution of the previously-increased cytokine expression with minor elevation of *CCL2* (1.8-fold-change as compared to the control) for CB-treated mice; also there was minor elevation of *IL6, IL12B, IL10* (1.8-fold-change as compared to the control), and *CCL2* (6-fold-change as compared to the control) for AgNP-treated mice (Figure 4). This indicates some continued activation of macrophages in both treatment groups, but also a distinct likelihood of continued monocyte recruitment for AgNP-treated mice (evidenced by CCL2 expression). There were no significant differences between treatment groups for protein concentration or phospholipid content; again consistent with maintenance of barrier function (Figure 5). However, there was a significant increase in SP-D content of AgNP-treated mice as compared to the control (Figure 6). Furthermore, there were no significant differences between treatment groups for capillary surfactometry (Table S1), consistent with phospholipid and SP-B findings. Analysis of respiratory mechanics revealed significant decreases in elastance and resistance spectra for AgNP-treated mice as compared to the control (Figure 7). The differences in spectra were demonstrated to be isolated to the low frequency end of the spectra for both elastance and resistance, indicating changes were occurring in the parenchyma (Figure 8). To determine if the results at 7 days post-instillation were due to the nano-scale properties of the AgNPs or ionic silver, a separate group of mice were dosed with silver nitrate solution. This treatment group demonstrated no signs of inflammation or significant differences in respiratory mechanics as compared to the saline control (data not shown).

These results indicate an acute inflammatory response to high dose CB instillations, without lung injury or significant changes in respiratory effort. While treatment with AgNPs led to a delayed immune response coupled with changes in surfactant composition and respiratory mechanics.

## Discussion

This study was focused on determining how the presence of silver within NPs would alter their effects on the lung. While high doses of non-metal NPs predictably led to acute inflammation (neutrophilia and macrophage activation), the same dose of AgNPs produced *no signs* of acute inflammation. Though the AgNP-treated mice lacked a classic inflammatory response, they experienced significantly increased work of breathing (at low and high doses of Ag-NPs). At 3 and 7 days post-instillation, inflammation resolved in the non-metal NP-treated mice, while the AgNP-treated mice experienced a delayed inflammatory response and a persisting decline in lung function. These findings confirm our hypothesis that there are inflammatory and lung function effects specific to the metal properties of AgNPs. We propose that these effects are due to modified airway epithelial cell function as evidenced by changes in surfactant production.

Non-metal NPs elicit a dose-dependent response displaying a lack of toxicological response at low doses while producing a classic irritant response of neutrophilic invasion and macrophage activation and high doses, similar to other work using CB particles (Wang et al., 2014). The AgNPs do not act in a similar dose-dependent manner. Neither at low nor high doses did AgNPs elicit an acute inflammatory response. This may be due to their lower propensity for aggregation and consequent interactions with components of the LLF. CB more easily self-aggregates, thus there is a higher probability of formation of larger agglomerates that can be easily phagocytosed by macrophages and trigger inflammatory pathways (as demonstrated by the increased expression of macrophage activation markers and neutrophilic invasion, Figures 3, 4). AgNPs readily maintain nanoscale form and are thus less likely to form large agglomerates than CB; however, they still have the capacity to interact with components of the LLF (Leo et al., 2013).

The acute respiratory mechanical effects of AgNPs are limited to changes in elastance in the low frequency portion of the inspiratory spectrum. These changes are consistent with alterations to the nature of the parenchymal tissue of the lung. This may be due to AgNPs becoming coated by lipid and surfactant protein from within the LLF. Such interactions have previously been reported to mediate the formation of NP strands in the presence of SP-B (Bakshi et al., 2008). This coating and strand formation could disrupt the maintenance of surface tension by the LLF, both by their presence but also by removing available surface-active material. Previously we have reported that 20 nm citrate-stabilized AgNPs have a greater propensity for aggregation and dissolution when lipid-coated as compared with both 20 and 110 nm PVP-stabilized AgNPs (Botelho et al., 2015).

Lipids comprise the largest percentage of macromolecules within the LLF [90%-dipalmitoyl phosphatidylcholine (DPPC), phosphatidylcholine (PC) and phosphatidylglycerol (PG)]; how NPs interact with this portion of LLF will likely determine further interactions with cells and proteins. The coating of AgNPs with lipid will increase the likelihood of interacting with alveolar macrophages, thus increasing lipid removal from the LLF. Loss of lipid could produce an increase in the work of breathing (Whitsett et al., 2010). While our capillary surfactometry results did not confirm a change in surface tension, this assay is limited in that it cannot detect localized changes within the lung. Lipid used in the capillary surfactometry assay is procured via a whole lung lavage. Localized changes that can be detected by the lung mechanics model may not be evidenced once examining the whole lung lavage, unless severe, wide-spread injury has occurred. Thus, the data presented here are supportive of a change in surface-active function of the LLF, but are not conclusive. Future studies focusing on using more sensitive assays, such as pulsating bubble surfactometry, would be valuable in determining potential changes to surface tension and their relative importance to lung function in the face of AgNP challenge.

The inflammatory response to high doses of non-metal NPs lessened at 3 days and resolved by 7 days post-instillation, which is consistent with an acute innate inflammatory response characterized by IL-1β and IL6 expression via inflammasome activation. However, mice dosed with AgNPs experienced a delayed inflammatory response. At 3 days post-instillation, AgNP mice demonstrated an invasion of lymphocytes, increasing macrophage count, and increased cytokine expression. This may represent a greater involvement of the acquired immune response system possibly via Th2 mechanisms, but as it also involves macrophage activation it seems reasonable to suppose there is inflammasome involvement. While the inflammatory pattern had worsened the initial tissue stiffness appeared to resolve 3 days after AgNP exposure. These data are best explained by reasoning that a cellular response has been mounted to compensate for the proposed change in LLF lipid content. Further it appears that the lung lining is signaling for cellular recruitment, and that increased lipid production by Type II epithelial cells has lessened the work of breathing. Type II epithelial cells are crucial to maintaining surfactant homeostasis; they are responsible for producing, secreting, and recycling surfactant proteins and lipid (Whitsett et al., 2010). Altered surfactant composition will trigger a Type II cell response to preserve both the immune and surface tension maintenance properties of the LLF. At 7 days post-instillation, while the inflammatory response had all but resolved in AgNP-treated mice, there was a significant decrease in both elastic and resistive components of the lung, specifically in the low frequency end of the spectra. This is potentially a result of an overshoot response by the pulmonary epithelium, in which the Type II cells have begun to over produce lipid. Additionally at 7 days, AgNP-exposed mice demonstrated an increase in SP-D, providing further evidence for changes in Type II cell function.

In conclusion, AgNPs appear to elicit metal-specific organ level responses within the lung. AgNP-surfactant interactions lead to reduced respiratory function and a delayed immune response, potentially via modified epithelial cell function. However, further studies are needed to examine long-term endpoints and to determine if the observed organ level effects resolve over time and how such responses are altered by chronic exposure. Continued exposure to AgNPs may lead to severe injury, prolonged inflammatory response, and remodeling of the lung tissue.

## Author contributions

DB conducted all of the *in vivo* experiments and prepared the first version of the manuscript; she was assisted by CM especially with functional analysis. SSa and SSc assisted with the surfactant exposure experiments and analysis. BL, SC, MR, and AP characterized the particles and did much of the *in vitr*o analysis. TT and KC supervised the work conducted in London and contributed significantly to the final version of the manuscript. JZ was the overall supervisor of the project and helped with the maunscript. EA-V assisted in the preparation of the manuscript and surfactant analysis. AG was the principal investigator and conceived of the study and was responsible for the final version of the manuscript.

### Conflict of interest statement

The authors declare that the research was conducted in the absence of any commercial or financial relationships that could be construed as a potential conflict of interest.
