# Supplementary material for: Exposure to Silver Nanospheres Leads to Altered Respiratory Mechanics and Delayed Immune Response in an in Vivo Murine Model
Source: Front Pharmacol. 2018 Mar 26;9:213. doi: 10.3389/fphar.2018.00213 (PMC5879457; doi:10.3389/fphar.2018.00213)
Supplement: Supplementary file 1 [file Presentation1.PPTX]

## Slide 1
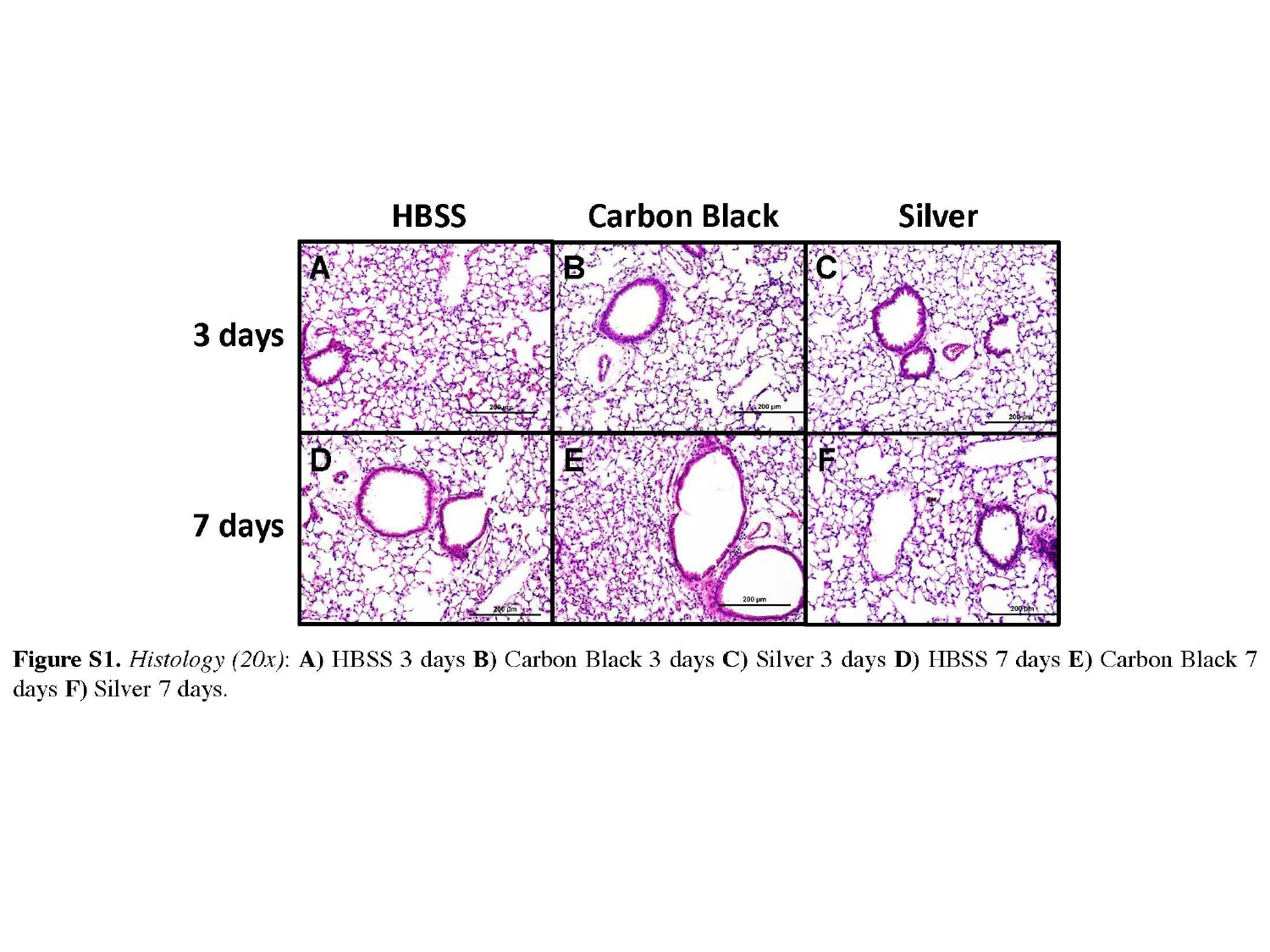

## Slide 2
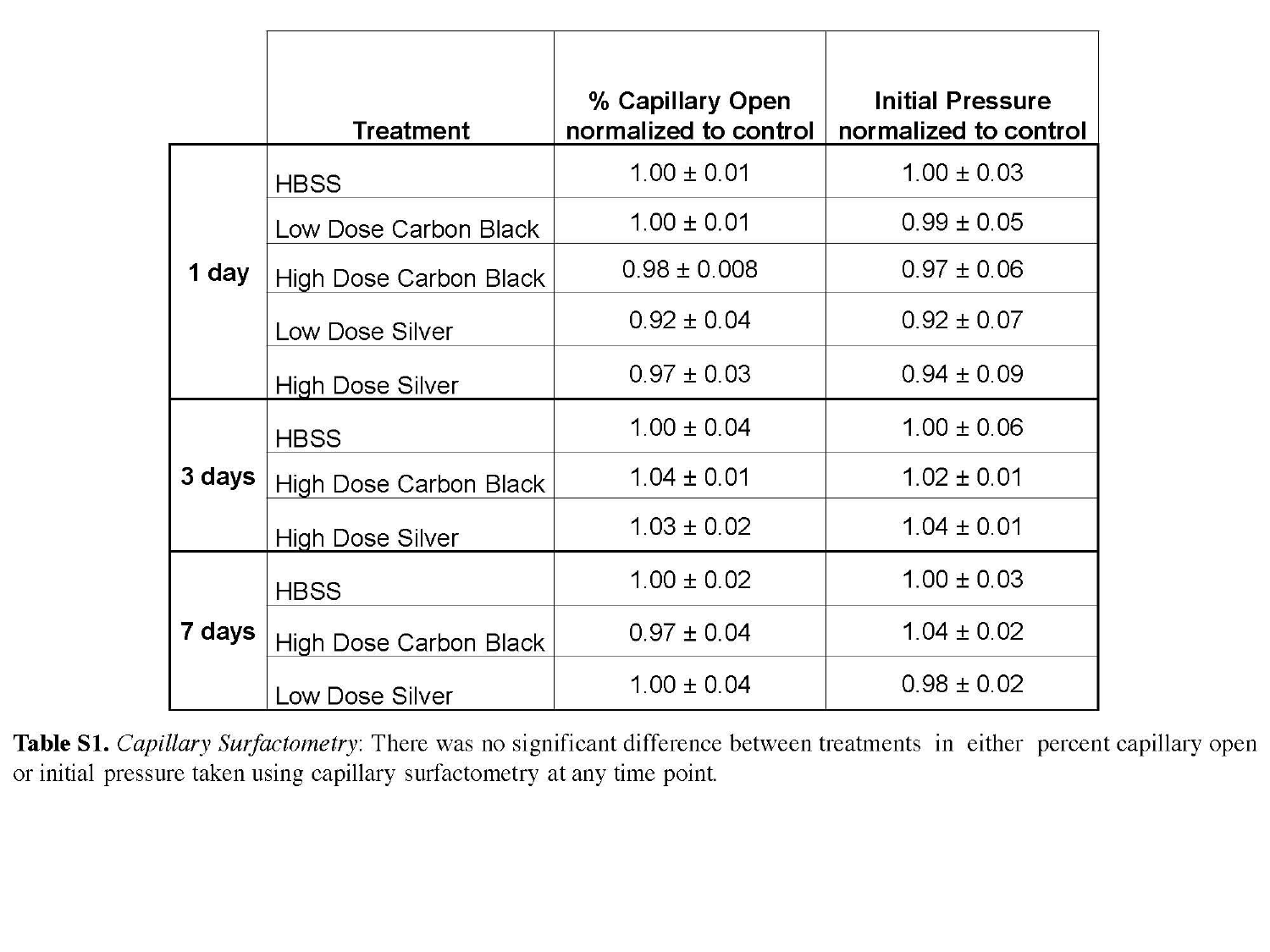

## Slide 3
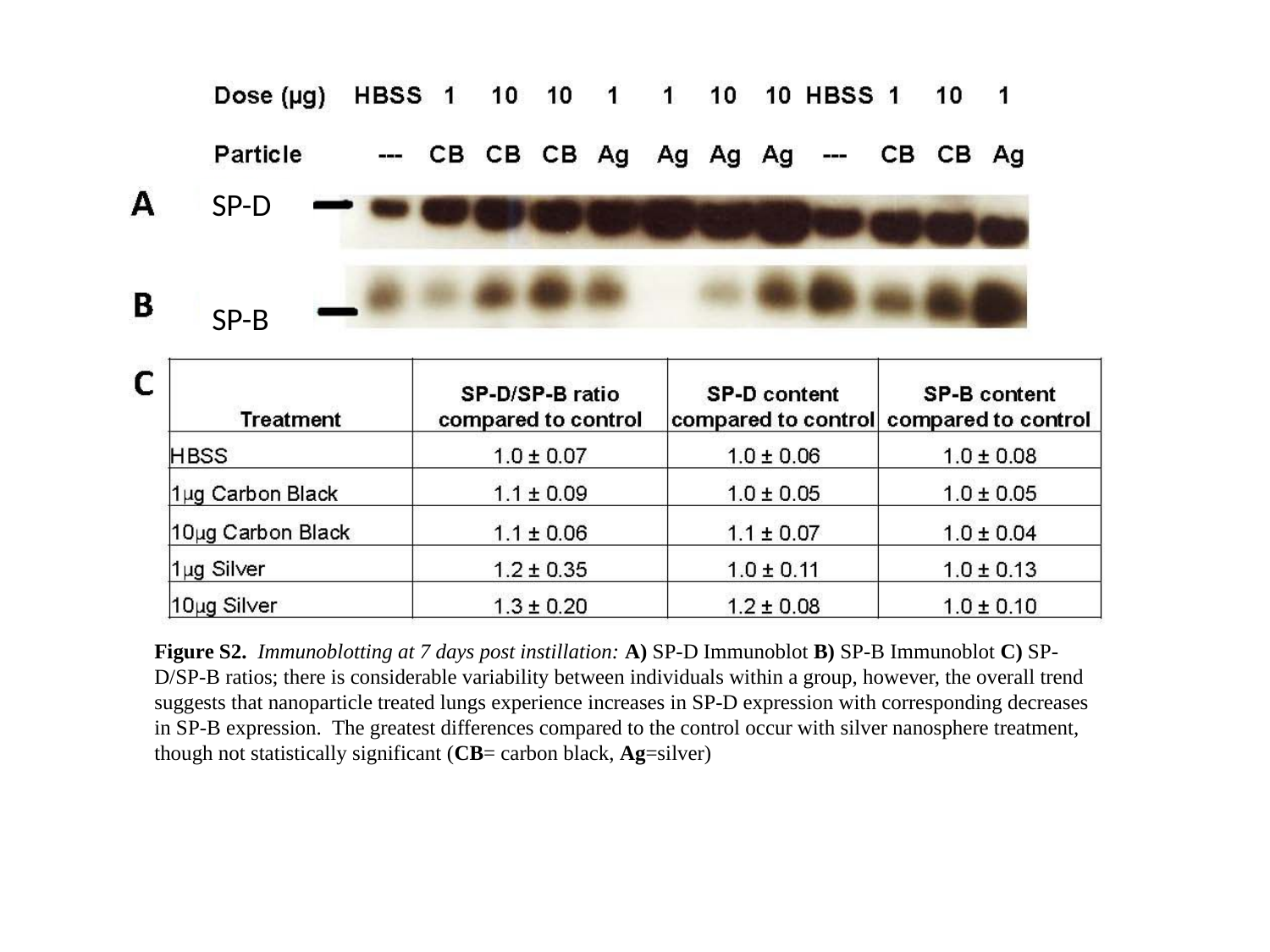

SP-D
SP-B
Figure S2. Immunoblotting at 7 days post instillation: A) SP-D Immunoblot B) SP-B Immunoblot C) SP-D/SP-B ratios; there is considerable variability between individuals within a group, however, the overall trend suggests that nanoparticle treated lungs experience increases in SP-D expression with corresponding decreases in SP-B expression. The greatest differences compared to the control occur with silver nanosphere treatment, though not statistically significant (CB= carbon black, Ag=silver)
